# Evaluation of *Marrubium vulgare* Growing Wild in Tunisia for Its Potential as a Dietary Supplement

**DOI:** 10.3390/foods10112864

**Published:** 2021-11-19

**Authors:** Marwa Rezgui, Mabrouk Basma, Nuno Neng, José Manuel Nogueira, Leila Bettaieb Ben-Kaab, Maria Eduarda Machado Araújo

**Affiliations:** 1 Faculty of Sciences of Tunisia, University Tunis El Manar, Tunis 2092, Tunisia; mabroukbasma1@gmail.com (M.B.); leila.bk@planet.tn (L.B.B.-K.); 2 Centro de Química Estrutural, Departamento de Química e Bioquímica, Faculdade de Ciências, Universidade de Lisboa, Campo Grande Ed. C8, 1749-016 Lisboa, Portugal; ndneng@fc.ul.pt (N.N.); jmnogueira@ciencias.ulisboa.pt (J.M.N.); mearaujo@fc.ul.pt (M.E.M.A.)

**Keywords:** antioxidant, essential oil, marrubiin, *Marrubium vulgare* L.

## Abstract

*Marrubium vulgare* L., known as horehound, is a widespread and widely known plant that is used in beer breweries and also as a traditional remedy in Tunisia. In this study, methanolic extracts of plants harvested from five different locations were investigated for their antioxidant activities using three assays (ferric reducing power, radical scavenging activity, and *β*-carotene-linoleic acid bleaching assay) as well as the total phenolic content. The mineral composition of the plant was also investigated concerning the following elements: Fe, Mg, Ca, Cu, Zn, Mn, K, and three heavy metals, Ni, Pb, and Cd. Marrubiin, the major bioactive diterpenoid lactone, was quantified by NMR in the samples. The essential oils were obtained by hydrodistillation, and their radical scavenging activity was investigated. The toxicity of essential oils was evaluated against *Artemia salina* (the brine shrimp larva). The essential oil showed a weak radical scavenging activity and low toxicity. Data obtained from the five different locations showed that the antioxidant activity, as well as the total phenolic and marrubiin content, were strongly affected by the harvest sites. The metal content in the samples showed differences with the harvest location, but there was always a great abundance of calcium, magnesium, and potassium.

## 1. Introduction

*Marrubium vulgare,* a plant from the Lamiaceae family whose genus includes about 97 species, is widespread along the Mediterranean Sea and in the temperate regions of the Eurasian zone [1]. This plant, commonly known as “horehound” in Europe or “Om Rubia” in Tunisia, grows wildly in dry sandy soils and wastelands and is frequently used as a raw material for herbal extracts and beverage industries. For instance, the plant has been used as a substitute for hops in beer breweries and is currently used to make herbal teas. In the old days, it was added to cooked vegetables, salads, and sauces. It is also very used in traditional medicine. *M. vulgare* is often an important source for the food and pharmaceutical industry. For example, only in India, there are 33 registered medicinal formulations containing white horehound [2]. *M. vulgare* possesses tonic, aromatic, stimulant, expectorant, diaphoretic, and diuretic properties. Its chemical composition includes phenyl propanoids, phenolic compounds, and diterpenic lactones, among other phytochemicals.

**Figure 1:**
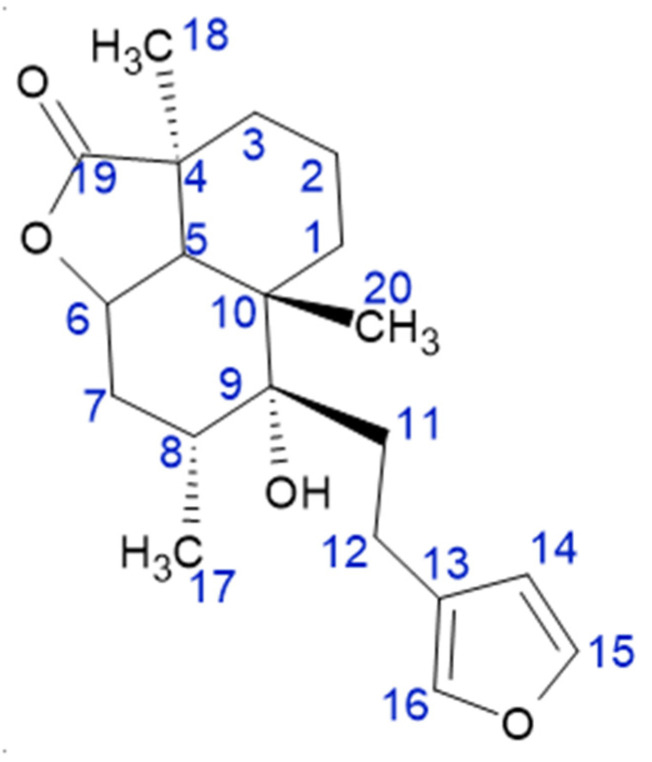
Chemical structure of marrubiin.

Earlier phytochemical investigation of *M. vulgare* led to the characterization of several labdane diterpenoids with marrubiin (Figure 1) as the main component [3]. Some authors [4] found an antispasmodic potential of marrubiin when administered to rabbit jejunum. Ca^2+^ antagonist potential, anticoagulant, and antiplatelet activities have also been detected [1,5]. Other authors [6,7] demonstrated that marrubiin exhibited a global inhibitory effect on different phlogistic agents. This labdane lactone also has the effect of increasing insulin secretion and LDL-cholesterol [8].

Other important secondary metabolites are the essential oils since they are, in some cases, highly bioactive, readily available in tropical countries, and economically viable. *M. vulgare* is moderately rich in essential oil, about 0.1%, according to Zarai et al. [9].

It is possible to distinguish between the Lamiaceae oil-rich and oil-poor species [10], being the *Marrubium* genus is considered as an oil-poor species. However, it has interesting bioactivities, and its use was recommended as a relaxant, expectorant, vasodilator, and antioxidant agent [10]. It is well known that the composition of essential oils is influenced by the presence of several factors, such as local, climatic, seasonal, and experimental conditions, and therefore, the chemical composition of *M. vulgare* essential oil from other origins, Lithuania, Czech Republic, has already been the subject of different studies [11,12,13]. However, to our knowledge, there is little information concerning the composition of this oil produced by plants growing wild in different geographic locations of Tunisia since this country contains very diverse habitats.

The phenolic profile of six wild-growing *M. vulgare* collected from different Tunisian arid and semi-arid zones showed marked interpopulational variability [14]. So, it is interesting to investigate if this variability also occurs with other environmental factors such as humidity and temperature. Regarding the mineral composition, the knowledge of the elemental content in medicinal plants and plants used as food supplements is also very important since the human body, to be healthy, needs a number of minerals that are necessary for growth, normal physiological functioning, and the maintenance of life. The mineral elements must be supplied by food, which led to a growing interest in the content of trace elements in herbs [15]. Although the number of scientific publications on *M. vulgare* has grown rapidly in recent years, to the best of our knowledge, there is no information on its inorganic elements important to the chemistry of life

Therefore, this work reports for the first time the intra-species variability of marrubiin and metal contents, essential oil composition and toxicity, and antioxidant activity of *M. vulgare* collected from five different zones of Tunisia.

## 2. Material and Methods

### 2.1. Reagents and Equipment

#### 2.1.1. Reagents

Anhydrous sulfate sodium, Folin–Ciocalteu reagent, gallic acid, potassium hexacyanoferrate, trichloroacetic acid, ferric chloride, ascorbic acid, 2,2-diphenyl-1-picrilhydrazyl (DPPH), 2,6-di-tert-butyl-4-hydroxytoluene (BHT), carvacrol, verbanone, linoleic acid, *β*-carotene, chloroform (HPLC grade), tween 40, silica gel, anhydrous sodium carbonate, nitric acid, and nitroperlchoric acid were provided by Sigma-Aldrich Quimica S.L., Lisboa, Portugal. Methanol, acetone, ciclohexane, ethyl acetate, and dimethylsulfoxide (DMSO) were from the Merck brand and provided by Laborspirit Lda, Loures, Portugal. Solvents were of analytical-grade quality.

#### 2.1.2. Equipment

UV-Vis spectra were acquired on Shimadzu 1603 double beam spectrophotometer (Izasa Scientific, Lisboa, Portugal). Fourier-transform infrared (FTIR) spectroscopy analyses were obtained in a Nicolet 6700 spectrometer (ThermoUnicam, Portugal) using KBr disk. Spectra were recorded in the 4000–650 cm^−1^ range at a resolution of 4 cm^−1^, averaging 128 scans per sample. Data were collected with Omnic 3.1 software (Thermo Fisher Scientific). ^1^HNMR (proton nuclear magnetic resonance) spectra were acquired in a Bruker Avance 400 apparatus (Bruker Optik GmbH, Ettlingen, Germany) at 400 MHz using chloroform-d as solvent. Mineral content was assayed by atomic absorption spectrophotometer with an Analyst 300 (Perkin Elmer, Norwalk, CT, USA). Gas chromatography-mass spectrometry (GC-MS) analyzes were carried out on an Agilent 6890 series gas chromatograph interfaced to an Agilent 5973 *N* mass selective detector (Agilent Technologies, Little Falls, DE, USA). Minerals were determined by an atomic absorption spectrophotometer with an Analyst 300 (Perkin Elmer, Norwalk, CT, USA).

### 2.2. Plant Material

*M. vulgare*, aerial parts, were harvested in their native ecosystems in Marsh 2016 from five different Tunisian regions (Table 1). The collected plant material consisted of total aerial parts.

The botanical identification of the plant was confirmed by Doctor Mouhiba Ben Nasri-Ayachi (member of botanical laboratory, “Faculty of Science of Tunis”), according to “Flore de la Tunisie” [16].

### 2.3. Extraction

*M. vulgare,* aerial parts, was air-dried in the dark at room temperature and then grounded to a fine powder. Extracts were obtained by mixing 2.5 g of the powder of each sample with 3 × 25 mL of methanol for 24 h at room temperature. Plant material was extracted three times, and the extracts were gathered, filtered through Whatman filter paper N4, and the solvent was removed by vacuum distillation. The yield of each extract was: Bizerte 61 ± 4.0 mg/g Dw; Boussalem 86 ± 8.7 mg/g Dw; Zaghouan 45 ± 7.0 mg/g Dw; Tunis 58 ± 9.8 mg/g Dw; Kasserine 84 ± 1.0 mg/g Dw.

To evaluate the marrubiin content, 2.5 g of dried plant was extracted with 3 × 25 mL of acetone. The extracts were then gathered. The solvent was removed by vacuum distillation and the residue was kept in the dark at 4 °C until analysis.

### 2.4. Distillation of Essential Oil and GC-MS Analysis Conditions

Essential oils were obtained by hydrodistillation of 50 g of dried plant material until 100 mL of the water-oil layer was obtained. Leaves and stalks were extracted separately. The aqueous layer was extracted with *n*-pentane, dried over anhydrous sodium sulfate, filtered, and the organic solvent was removed by vacuum distillation at room temperature. Essential oils were stored at 4 °C until analysis. Gas chromatography-mass spectrometry (GC-MS) analysis was performed as previously described [17].

### 2.5. Toxicity to the Aquatic Microcrustacean Artemia Salina

The toxicity of essential oils was determined in vitro, as was described in a previous work [18]. Artificial salty water (larvae medium) was obtained by dissolving 1.9 g marine salt from the Ria Formosa Natural Park in Algarve, Portugal, in 250 mL of distilled water. The pH was adjusted to 9.0 using Na_2_CO_3_ to avoid the risk of death of *A. salina* larvae by a decrease in pH during incubation.

After 48 h of incubation at 25 °C under aerobic conditions, 1 mL containing 15 larvae was transferred with a micropipette to a test tube, 0.5 mL of essential oil dissolved in DMSO (2% *v*/*v*) were added, and artificial saltwater was added to a final volume of 5 mL. In the control tubes, extracts were replaced by distilled water.

The 24 h live larvae were counted, and the mortality was corrected using the Abbott formula:

Corrected mortality (%) = [1 − (nt/nc)] ∗ 100(1)

where nt = treated larvae population, nc = control larvae population. Tests were performed in triplicate.

### 2.6. Determination of Total Phenolic Compounds

Total phenolic compounds content was determined by colorimetric assay, using the Folin–Ciocalteu reagent [19] and gallic acid as a standard. Briefly, 3 mg of each crude methanol extract was dissolved in 1 mL of methanol and then mixed with 4.5 mL of distilled water. Then 1 mL of Folin–Ciocalteu reagent was added. The mixture was shaken and allowed to stand for 5 min. After 3 min, 3 mL of a 2% solution of sodium carbonate was added and mixed thoroughly. After incubation in the dark for 2 h with intermittent shaking, the absorbance (Abs) was measured at 760 nm. The concentration of total phenolic compounds was determined as milligram gallic acid equivalents per gram of extract using a calibration curve obtained with gallic acid, y = 0.922x (R^2^ = 0.97), where y is the absorbance of the methanolic test solution, gallic acid, or methanol extract and x is the concentration of gallic acid solution. Results were computed and presented in Table 2 as mg GA equivalent/g dry plant (DW). The assay was determined in triplicate for each extract.

### 2.7. Antioxidant Activity

#### 2.7.1. Fe^3+^−Fe^2+^ Reduction

The Fe^3+^−Fe^2+^ reduction was monitored by measuring the formation of Prussian blue pigment, KFe[Fe(CN_)6_] at 700 nm [20]. In brief, 1 mL of methanol extract with different concentrations was mixed with 2.5 mL phosphate buffer (0.2 mol/L pH = 6.6), and 2.5 mL of a 10 g/L potassium ferricyanide K_3_Fe(CN)_6_ solution. After 30 min at 50°, 2.5 mL of a 100 g/L aqueous trichloroacetic acid (TCA) solution was added, and the mixture was stirred with a glass rod. Finally, a 2.5 mL aliquot was added to 2.5 mL ultra-pure water and 0.5 mL of a 1 g/L FeCl_3_ solution, and the absorbance (Abs) was measured at 700 nm. The iron (Fe^3+^) reductive capacity was calculated by a standard ascorbic acid graph, y = 8.215x + 0.03 (R^2^ = 0.95), where y is the absorbance of the test solution, ascorbic acid, or methanol extract solution, and x is the concentration of the ascorbic acid solution. Results are expressed as ascorbic acid equivalents (AscAE; mmol ascorbic acid/g sample) and presented in Table 2. The assay was determined in triplicate for each extract.

#### 2.7.2. DPPH Radical Scavenging Activity

The DPPH (2,2-diphenyl-1-picrilhydrazyl) radical scavenging activity of the methanol extracts was determined using the method described by Mata et al. [21]. Methanol extract concentration providing 50% inhibition (EC_50_) was calculated using regression analysis in MS Excel. The assay was performed in triplicate for each extract. Results are presented in Table 2.

Since essential oils are poorly soluble in methanol, the ability of *M. vulgare* oil to scavenge free radicals was assayed by an optimized method. Briefly, 100 µL of essential oil, 300 µL DMSO, 1600 µL methanol, and 1 mL of DPPH 200 µM were mixed. A control solution was prepared containing all reagents and having 100 µL of DMSO instead of sample. The mixture was shaken vigorously and allowed to stand for 30 min in the dark at room temperature prior to measuring the absorbance spectrophotometrically at 517 nm. The radical scavenging activities of the tested samples were expressed as the percentage extinction of the DPPH radical and calculated according to the usual formula:

E(%) = [(A_control_ − A_sample_)/A_control_] × 100 (2)

where A_Control_ is the absorbance of the control solution (containing all reagents except the test extract), A_Sample_ is the absorbance of the sample containing the tested extract.

For comparative purposes verbanone, carvacrol, and a solution of butylhydroxytoluene (BHT) (c = 1 mg/mL) were tested and used as standards. Tests were performed in triplicate for each extract. Results are presented in Table 3.

#### 2.7.3. β-Carotene/Linoleic Acid Assay

Lipid antioxidants were evaluated by measuring the inhibition of the formation of volatile organic compounds and conjugated diene hydroperoxides arising from linoleic acid oxidation. The method described by Khadri et al. [22] was used with a slight modification. A stock solution was prepared, dissolving 20 mg of β-carotene in 10 mL chloroform (HPLC grade). A total of 1 mL of this solution was mixed with 25 µL of linoleic acid and 200 mg of tween 40 into a 500 mL round-bottomed flask. Chloroform was completely evaporated using a vacuum evaporator. Then 50 mL of aired distilled water was added to the flask with vigorous shaking. A total of 2.5 mL aliquots of this reaction mixture were dispersed to a series of tubes containing 300 µL of methanol extract with a final concentration of 1.6 mg/mL and were further shaken. The reaction mixture was immediately transferred to the UV cells and placed in a thermostatized UV-Vis unit at 50 °C, and readings were taken automatically at 470 nm with 2 min interval for 120 min. After that time, the color of β-carotene had disappeared. Two blanks, one containing only water and the other containing the same volume of methanol instead of the extract solution, were also prepared.

The absorbance of each sample at 50 °C by the end of the 120 min reaction was registered. The antioxidant activity (AA) of the extracts under investigation was expressed as:

AA(%) = {1 − [A(t = 0) − A(t = 120)/A0(t = 0) − A0(t = 120]} ∗ 100(3)

where AA is the antioxidant activity, A(t = 0) is the absorbance of the sample in the investigation at 0 min, A(t = 120) is the absorbance of the same sample after 120 min, A0(t = 0) and A0(t = 120) is the absorbance of the positive control (methanol without extract) at t = 0 min and t = 120 min, respectively. Results are presented in Table 2.

### 2.8. Isolation of Marrubiin and Quantification of Marrubiin Labdanoids

A total of 50 g of *M. vulgare* (dry material) were percolated with acetone. A total of 18 portions were collected and then analyzed by TLC in silica gel plates using cyclohexane-ethyl acetate (6:4) as eluent. Portions having the same chromatographic profile were gathered to obtain 5 fractions. Fraction 2 (1.01 g) was washed with petroleum ether to remove oil and coloring matter yielding 0.707 g of residue. To separate marrubiin, the crude material was purified by flash chromatography on silica gel using an increasing proportion of ethyl acetate in cyclohexane. Fractions containing marrubiin were pooled and crystallized from methanol to afford 131 mg of the compound. The identity of marrubiin was checked by FTIR, ^1^H, and ^13^C NMR. Spectroscopic data were identical to the literature [23]. To quantify the amount of marrubiin in different samples, a calibration curve was performed with known amounts of marrubiin, according to the method previously described [23]. The integrated signal area (I) of the peak at 7.38 ppm corresponding to H-15 (Figure 1) was used to obtain a calibration curve:

I_7.38ppm_ = 4∙10^6^ [marrubiin] − 3∙10^6^ (R^2^ = 0.98) (4)

A similar calibration curve was obtained for the peak at 6.29 ppm corresponding to H-14 (Figure 1).

To determine the quantity of marrubiin, 10 mg of each acetone extract was dissolved in 1 mL of deuterated chloroform, and all spectra were recorded in the same conditions. Results were computed and presented in Table 4 as the content of marrubiin (g) per g of dry material (DW).

### 2.9. Determination of Mineral Contents

All the plastic and glassware were cleaned by soaking with contact overnight in a 10% nitric acid solution and then rinsed with deionized water. All samples were treated in an identical manner. For acid digestion, 25 mg of dry plant material was weighted into a pre-cleaned beaker, 5 mL of nitroperchloric acid was added, the beaker was covered with a watch glass, and the sample boiled gently on a laboratory hot plate until digestion was complete. This process took approximately 1 h. The digested sample was then allowed to cool before being transferred quantitatively into clean 30 mL volumetric flasks, and the volume was completed by the addition of nitric acid (7%). Tests were performed in triplicate. Results are presented in Table 5.

### 2.10. Statistical Analysis

Statistical analysis was performed with the SPSS 18.0 software (SPSS Inc.). Statistical comparisons were made with one-way ANOVA followed by Tukey multiple comparisons. The level of significance was set at *p* < 0.05. Correlations between phenol content, marrubiin, and antioxidant activity were achieved by Pearson correlation coefficient (r) at a significance level of 99% (*p* < 0.01)

## 3. Results and Discussion

### 3.1. Plant Material and Sampling

*M. vulgare* (white horehound or common horehound) is popularly named “Om Rubia” in Tunisia. It grows wildly in all bioclimatic zones and geographical regions except in the extreme South of the country [14]. During an investigation about the importance of folk medicine performed by some authors [24], a questionnaire concerning the use of herbal medicines has been proposed to patients followed in the National Institute of Nutrition of Tunis. Of the two hundred patients that answered the questionnaire, 10.9% used *M. vulgare* as a traditional remedy.

In this study, *M. vulgare* was harvested from different sites in Tunisia in order to form a scientific base of the traditional use of this crop and to study the possible diversity between plant populations, which can possibly be linked to the change of bioclimatic conditions (Table 1).

### 3.2. Chemical Composition and Toxicity of Essential Oils

#### 3.2.1. Chemical Composition of Essential Oils

The components of the essential oils were identified by their percentage and their retention indices (RI) relative to (C8–C22) *n*-alkanes. The identity of each compound was determined by comparison of its spectra with the Wiley library spectral data bank (G1035B; Rev D.02.00; Agilent Technologies). Table 6 presents the components with a percentage above 0.1 in order of their elution on the HP-5 column. Components that are present in a percentage lower than 0.1 or as trace are not listed. Our data confirm that *M. vulgare* is a Lamiaceae poor oil species and that no leaves or stems are more reached in aromatic compounds. The composition of the oil varies between the different locations. Essential oil of *M. vulgare* collected in Kasserine provided the poorest yield, and data is not presented. Essential oils obtained from the leaves of plants collected in Boussalem and Tunis contain more diverse components being eugenol, the more abundant chemical, 15.89% and 8.06%, respectively. The richest oils in terpenoids are those from the leaves of plants from Boussalem, Zaghouan, and Tunis. Interestingly, the steams of plants from Zaghouan contain a relatively large amount of marrubin, 6.67%. Regarding the previously reported composition of *M. vulgare* by Zarai et al. [9], it is interesting to point out that there are important qualitative and quantitative differences indicating that the environmental factors strongly influence its chemical composition.

#### 3.2.2. Toxicity against Artemia Salina

It is currently a tendency to limit the use of laboratory animals in toxicological tests. The method that uses brine shrimp (*Artemia salina*), a commercially available crustacean whose larvae are sensitive to a variety of substances, is an easy, low-cost, and quick bioassay for predicting the toxicity of plant extracts and guiding their phytochemical fractionation [18]. This is of particular importance in developing countries where a large percentage of the population relies on the use of crude medicinal plant extracts to meet their health care needs [25].

Essential oils were tested at 2% (*v*/*v*). The toxicity of essential oil from the leaves of plants collected at Zaghouan is not presented here since the amount obtained did not allow to obtain a result with the same accuracy. However, in a single experiment, it was of the same magnitude as the others. Results are presented in Table 7.

All essential oils exhibited low toxicity against *A. salina* and demonstrated high cell viability following exposure for 24 h. Among the evaluated samples, essential oils from Bizerte were the least toxic.

### 3.3. Total Phenolic Compounds

Polyphenolic compounds are the major groups of chemicals that have the ability to eliminate radical species preventing the propagation of oxidative chain reactions and acting, consequently, as primary antioxidants or free radical terminators [26], so it is very important to quantify polyphenols in different samples.

Genetics and environment are the main reason why plants vary widely in their phenolic composition and content [27]. There are numerous studies demonstrating that intraspecific variability between populations from different sites may result in differential antioxidant properties [28,29]. Our results showed that the total polyphenol contents of all samples were quite important, ranging from 11.44 ± 0.12 mgGAE/g DW (469.03 mg GAE/g methanol extract) in Bizerte plants to 31.89 ± 0.35 mgGAE/g DW (927.33 mg GAE/g methanol extract) in Boussalem, as is depicted in Table 2 and are in accordance with those studies showing the effect of provenance on the phenolic content of *M. vulgare*. Thus, our results thus confirm that the edaphic conditions are one of the major factors playing a role in the differences in the chemical composition of metabolites and bioactivities of the plants, as the above example illustrate since plants from regions with a similar inferior humid bioclimatic stage (Boussalem and Bizerte) are quite different in their total phenolic content.

A similar result had been obtained by other authors [29] that, studying two closer Tunisian provenances of another plant, *Tamarix gallica*, from two arid regions, superior and inferior bioclimatic stages with different edaphic factors, especially soil salinity, found significant differences in the total polyphenols content and antioxidant activities.

### 3.4. Antioxidant Activity

The antioxidant activity is a complex property that cannot be evaluated by a single test, furthermore when the material to be checked are plant extracts whose chemical composition is usually complex. To solve this problem, various methods were developed to evaluate the effectiveness of these compounds. In this work, three methods have been used, the ferric reducing power that gives information about the total antioxidant activity, scavenging of the stable DPPH radical was used as a model of oxygen and nitrogen radicals, and the test of the bleaching of β-carotene was used as a model for inhibition of the formation of lipid peroxides.

#### 3.4.1. Ferric Reducing Power

The ferric reducing antioxidant power established by Benzie et al. [30] was used not only to assess the ferric reducing ability of biological fluids such as blood and plasma but also the antioxidant capacity of various dietary agents. It is assumed in this test that all antioxidant compounds (reductants) able to reduce the Fe^3+^/Fe^2+^ system are also able to reduce ROS (reactive oxygen species) and RNS (reactive nitrogen species). Some complexes were used instead of the initially established ferric-tripyridyltriazine complex (Fe^3+^/TPTZ) [31]. In this work, we used the ferricyanide reagent K_3_Fe(CN)_6_ that forms the Prussian blue compound KFe[Fe(CN_)6_] [31], and we expressed the antioxidant activity in ascorbic acid equivalents (Asc AE).

Regarding the reducing power activity based on the reduction in Fe^3+^ into Fe^2+^ in the presence of the methanol extracts of *M. vulgare,* results revealed the potential reducing activities of plant extracts. In fact, all extracts investigated were able to promote the reduction in ferric to ferrous ions (Table 2). Like what was observed with the total phenolic content, extracts obtained from plants collected in Zaghouan presented the highest reducing power (225.87 ± 0.01 Asc AE) and those from Tunis the lowest one (45.99 ± 0.11 Asc AE) (Table 2).

#### 3.4.2. DPPH Radical Scavenging Activity

In the present study, the ability to scavenge free radicals has been evaluated using the stable DPPH radical (Table 2). According to Amarowicz et al. [32], the DPPH test has the advantage of not being affected by certain side reactions, such as metal ion chelating and enzyme inhibition.

Results revealed that all the investigated methanol extracts demonstrated a radical scavenging activity (RSA) and depicted a large and significant variability: the EC_50_ varied from 30.66 ± 0.03 µg.mL^−1^ (Zaghouan) to 2884.66 ± 0.01 µg.mL^−1^ (Tunis). In fact, the EC_50_ values of the extract of Zaghouan surpass those found for the other provenances and those reported by other authors for *M. vulgare* of the close country Algeria, 522 µg/mL [33]. The essential oils obtained from leaves and stems of *M. vulgare* collected in four accessions were also tested by the DPPH assay. Since essential oils are poorly soluble in methanol, the ability of these essential oils to scavenge free radicals was assayed by an optimized method as described in experimental. At the concentration tested, 33 µL. mL^−1^, all of them showed a weak RSA (Table 3). The essential oil obtained from the leaves of *M. vulgare* collected in Tunis was the most active (70.68%). The RSA of these oils was in general higher than the RSA of the pure monoterpenes used as standards, carvacrol (27%) and verbenone (33.73%) tested at the same conditions. They were also higher than a solution of a synthetic antioxidant BHT that, at a concentration of 1 mg/mL, presented only 24.35% activity. The higher ability of essential oils obtained from Tunis and Boussalem plants to act as an electron donor followed by proton transfer into the transformation of DPPH^•^ to its reduced form DPPH-H was thought to be due to the presence of a higher amount of the oxygenated phenyl propanoid eugenol.

#### 3.4.3. Antioxidant Activity in the β-Carotene–Linoleate Model System

The antioxidant activity can also be expressed as the ability to inhibit the oxidation of linoleic acid, which simulates at the same time the ability to inhibit the oxidation of the lipid membrane components and the formation of the hydroperoxide conjugated diene due to the linoleic acid oxidation. The presence of an antioxidant protects linoleic acid from being oxidized, thus avoiding the formation of linolyl peroxides that can attack β-carotene and, the result is that the test solution maintains its characteristic yellow-orange color. Thus, a slow degradation rate of β-carotene indicates the presence of lipophilic antioxidants, which can hinder the extent of its destruction by “neutralizing” the linoleate free radical (i.e., using its redox potential) and any other free radicals formed within the system [34]. Our results showed that the five methanolic extracts tested exhibited different capacities to inhibit the bleaching (Table 4) with the following order: Zaghouan (46.41%) >Kasserine (38.99%) > Tunis (37.03%) > Bizerte (36.33%) > Boussalem (2.38%).

### 3.5. Marrubiin Labdanoids Content

The biosynthesis of some compounds, such as terpenoids, play a role in the plant–environment interaction and its extent depend on the alteration between photosynthetic and respiratory processes, functions that are conducted by intrinsic factors (genotype and ontogenesis), and extrinsic factors such as light, temperature, and nutrients that act in the plant [35].

One of the major constituents of *M. vulgare* is marrubiin, a widely known diterpenoid lactone that constitutes the bitter principle of many medicinal plants of the Lamiaceae family [3,8]. Extensive pharmacological researches revealed that the diterpene marrubiin presents a large number of activities, namely gastroprotective [1], cardioprotective [36], antispasmodic [1], and antinociceptive [37]. The anti-diabetic activity of marrubiin was also proved in vivo using an obese rat model [8], which resulted in an increase in insulin secretion. In this work, marrubiin was isolated from acetone extract of wild *M. vulgare* collected from the region of Boussalem. FTIR, ^1^H, and ^13^C NMR were used to confirm the identity of isolated marrubiin by comparison with literature data [23]. Investigation of proton spectra (data not shown) showed three well-separated peaks at 6.29 and 7.25 and 7.38 ppm corresponding to protons H-14, H-16, and H-15, respectively, of the furan ring (Figure 1). Besides marrubiin other marrubiin labdanoids containing the furan ring, such as marrubenol peregrinin, 11-oxomarrubiin, and 12(S)-hydroxymarrubiin, may be present in small amounts [38]. Regarding the two former compounds, it is not expected that the chemical shift of the furan hydrogen atoms is sensitive to differences in the oxygenation state at positions in the opposite site of the molecule, as is the case of peregrinin and marrubenol or to the presence of an oxo group in the C-12. So, the intensity of the peaks at 7.38 ppm and 6.29 ppm were used to quantify, in the acetone extracts, the whole amount of marrubiin and the other minor marrubiin labdanoids aforementioned [38]. The peak at 7.25 ppm was not used for quantification because it was close to the peak of chloroform, and it could result in erroneous results. Results were expressed in mg of marrubiin per gram of dry material (DW).

From Table 4, it can be seen that marrubiin content was different in plants collected at different localizations: Tunis and Kasserine contained the highest amount (5.98 and 5.14 mg/g DW, respectively), Boussalem and Bizerte presented medium values (2.60 and 1.65 mg/g DW, respectively), and, unexpectedly, marrubiin was not found in *M. vulgare* collected from Zaghouan.

### 3.6. Correlation between Polyphenols Content, Marrubiin, and Antioxidant Activities

In general, most phenolics exhibit some degree of antioxidant activity. In general, extracts with a high antioxidant capacity would show a high phenolic content as well. Moreover, polyphenols found in plant extracts are considered the main bioactive compounds with antioxidant activity. Thus, correlation coefficient (r) was calculated in order to estimate the correlation between TPC, marrubiin, and antioxidant activities (RSA, ferric reducing power, and bleaching of beta carotene). In this work, the correlation coefficient between phenolics and both DPPH• scavenging activity and ferric reducing power was very significant with R^2^ values of −0.599 and 0.592 (*p* < 0.01), indicating that polyphenolics may play an important role in free radical scavenging. However, a weak correlation was found between TPC and bleaching beta carotene, which can be explained by the f different mechanisms of each test or assuming that different polyphenols acted as an antioxidant in the three assays. Results also showed that the content of marrubiin correlates with the DPPH• scavenging activity (0.698, *p* < 0.01), which is a further confirmation of the RSA of marrubiin. [38]

### 3.7. Metal Content

Mineral composition is an important factor for qualitatively evaluating the nutritional value of a plant and the potential use for different products. Seven elements (Fe, Mg, Ca, Cu, Zn, Mn, and K) and three heavy metals (Ni, Pb, Cd) were determined in the six samples of *M. vulgare*. The mean concentrations of various metals in *M. vulgare* are presented in Table 5. From this data, it was revealed that all the metals were accumulated to greater or lesser extents by all five samples studied. Here, we note that our focus is not only to compare but rather to explore the mineral composition in *M.vulgare* and to discuss their importance in human health. Minerals are dietary requirements for humans and exert various physiological effects. Due to a deficiency of these minerals in the human diet, most of these minerals are often taken as supplements. Only in India, there are 33 registered medicinal formulations containing *M. vulgare* [2], but where it is undoubtedly known is in the USA. In 2015, *M. vulgare* preparations were the best-selling herbal dietary supplements, reaching approximately USD $106 m in retail sales [39]. Iron is an important metal in the production of hemoglobin in blood and myoglobin in muscles. The human body requires Fe for the construction of several proteins in the blood, and this metal is also found in many proteins in the body where it performs many roles. Iron content was investigated, being the plants from Kasserine the richest in this element (9104 ± 6.26 ppm) and those from Boussalemthe poorest (208 ± 1.33 ppm). The abundance of Mg and Ca in *M. vulgare* was in agreement with previous reports, which suggested that these two metals represent the most abundant metal constituents of many plants [40,41]. The former is very important in human health since high amounts of calcium are required in the formation and maintenance of bones, teeth, muscle system, and in heart functions [42]. In fact, the plants contained large amounts of these metals, having plants from Bizerte the highest value in magnesium, 12,840 ± 3.91 ppm, and those from Kasserine, 7672 ± 0.38 ppm, the smallest. Regarding the content of calcium, the highest value was found in Zaghouan, 55,760 ± 30.91 ppm, and the smaller in Tunis, 46,160 ± 1.23 ppm. Potassium, another important metal, ranged from 20,800 (Boussalem) to 8400 ppm (Zaghouan).

Copper and zinc are two metals absolutely required in the human diet since they exhibit a wide range of biological functions such as components of enzymatic and redox systems [43]. *M. vulgare* contained copper that ranged from 17.13 ± 0.03 to 55 ± 09 ppm and also zinc that ranged from 1309.23 ± 0.77 to 531 ± 0.3 ppm. The presence of manganese in plants may be correlated with therapeutic properties against diabetic and cardiovascular diseases. This element was also found in *M. vulgare,* and values ranged between 33 ± 0.09 (Tunis) and 5.31 ± 0.01 ppm (Bizerte).

Concerning the heavy metals, lead was not present, values for the cadmium concentrations were in the range of 8.33 ± 0.05 ppm (Kasserine) and 13 ± 0.0 ppm (Bizerte), and the nickel concentrations varied from 22.33 ± 0.07 ppm (Kasserine) and 122.32 ± 0.08 ppm (Tunis). Our research supports the hypothesis that many factors such as the genetics of the plant, soil conditions, rainfall, and even altitude play an important role in the variation of nutrient element and metal content in plants. Our present study actually underscores the potential utility of *M. vulgare* in home recipes or herbal medicine that may be of interest to consumers.

## 4. Conclusions

*M. vulgare* is sold as a food supplement in many counties, but the botanical origin is never indicated. In addition to nutritional substances, food supplements also provide other substances that have a positive impact on human health. Our investigation of *M. vulgare* collected in five different provenances in Tunisia showed that, whatever location plants were collected, these plants had important phenolic content and antioxidant activities. However, values obtained indicated that these activities were affected by environmental constraints. These constraints were notorious in the chemical composition of essential oils and, consequently, its radical scavenging activity. Fortunately, although its composition varied, the toxicity of all essential oils was always low.

The results also showed that changes in the habitat environment greatly influence the production of important bioactive metabolites, namely marrubiin, that unexpectedly were not detected in one location. It was also confirmed that the plant contained a large amount of minerals, especially calcium, magnesium, and iron. Although the global amount of the investigated mineral was of the same magnitude, the difference in the amount of some metals such as nickel, copper, and zinc there were five- to two-fold more. A huge difference, 43 times more, was found in the content of iron between Boussalem and Kasserine.

Finally, since food supplements are attracting great interest by the consumer that uses them to maintain and treat minor health problems, our study highlights the importance of this plant, the importance of the detection and quantification of the bioactive phytochemicals, and furthermore, the importance of the knowledge of the environment were plants used in food supplements or has medicinal ones grow because it greatly affects the production of bioactive metabolites and consequently its effect.

## Figures and Tables

**Table 1:** Climatic characteristics of the studied localities where *M. vulgare* was harvested.

Harvest Site	Bioclimatic Stage ^a^	Latitude	Longitude	Altitude (M)	Rainfall (Mm/Year)	Mean Temperature (°C)
Bizerte	Inferior humid	37°16′00″ N	9°52′00″ E	37	442	19
Boussalem	Inferior humid	36°36′40″ N	8°58′11″ E	141	404	18.4
Zaghouan	Superior semi-arid	36°21′07″ N	10°06′43″ E	800	617	17.7
Tunis	Subhumid	36°47′51″ N	10°09′57″ E	40	446	16
Kasserine	Inferior semi-arid	35°10′00″ N	8°50′00″ E	656	239	18.1

^a^ Emberger’s pluviothermic coefficient: Q2 = 2000 × P/(M_2_ − m_2_) where P is the mean of annual rainfall (mm), M is the mean of maximum temperature (K) for the warmest month, and m is the mean of minimum temperature (K) for the coldest month. P, M, and m values were calculated as the average for the period from 2014 to 2016.

**Table 2:** Phenolic content and antioxidant activity of *M. vulgare*.

Harvest Site	TPC (mg GAE/gDW)	Reducing Power Fe^3+^/Fe^2+^ (Asc AE)	DPPH Scavenging (EC_50_ µg.mL^−1^)	Bleaching of Beta Carotene (%) *
Boussalem	11.44 ^a^ ± 0.12	149.72 1 ^b^ ± 0.00	780.54 ^b^ ± 1.05	2.38
Bizerte	31.89 ^c^ ± 0.35	125.86 1 ^b^ ± 0.50	1292.78 ^c^ ± 4.94	36.33
Tunis	16.81 ^c^ ± 0.14	45.99 1 ^a^ ± 0.11	2884.66 ^d^ ± 0.01	37.03
Zaghouan	13.61 ^ab^ ± 0.01	225.876 1 ^d^± 0.01	30.66 ^a^ ± 0.03	46.41
Kasserine	24.56 1 ^b^ ± 0.01	199.82 1 ^c^ ± 2.78	319.36 ^a^ ±1.05	38.99

Each value represents the mean ± SD (*n* = 3); TPC, total phenolic compounds; GAE, gallic acid equivalents; DW, dry weight; Asc AE equivalents, mg ascorbic acid/g sample; * Concentration tested = 1.6 mg·mL^−1^. Means followed by the same letter are not different according to ANOVA (analysis of variance) (* *p* < 0.05), a > b > c > d.

**Table 3:** Radical scavenging activity (RSA) of essential oils evaluated by the DPPH test.

Harvest Site	RSA (%) ^1^	
	Leaves	Stems
Boussalem	57.74 ^f^ ± 0.06	35.96 ^f^ ± 0.07
Bizerte	22.93 ^b^ ± 0.08	32.43 ^d^ ± 0.08
Tunis	70.68 ^g^ ± 0.06	27.55 ^c^ ± 0.07
Zaghouan	20.02 ^a^ ± 0.09	ND ^2^
Verbanone	33.73 ^e^ ± 0.02	
Cravacrol	27.00 ^d^ ± 0.04	
BHT ^3^	24.35 ^c^ ± 0.35	

^1^ Each value represents the mean ± SD (n = 3); ^2^ Not determined; ^3^ BHT: Butylhydroxytoluene; Concentration of EO = 33 µL. mL^−1^. Means followed by the same letter are not different according to ANOVA (analysis of variance) (*p* < 0.05), a > b > c > d > e > f > g.

**Table 4:** Marrubiin content of *M.vulgare* from different locations *.

Harvest Site	Marrubiin Content (mg/g DW ^1^)	
	Peak 1 ^2^	Peak 2 ^3^
Boussalem	2.60 ^b^ ± 0.28	2.73 ^b^ ± 0.34
Bizerte	1.65 ^a^ ± 0.33	1.82 ^a^ ± 0.34
Zaghouan	nd ^4^	nd ^4^
Tunis	5.98 ^c^ ± 0.77	6.84 ^d^ ± 0.98
Kasserine	5.14 ^c^ ± 0.01	5.5 ^c^ ± 0.01

* Mean ± SD (*n* = 3); ^1^ DW—dry weight; ^2^ calculated using the area of the peak at 7.38 ppm in the ^1^H NMR spectra; ^3^ calculated using the area of the peak at 6.29 ppm in the ^1^ H NMR spectra. ^4^ nd—not detected. Means followed by the same letter are not different according to ANOVA (analysis of variance) (* *p* < 0.05), a > b > c > d.

**Table 5:** Mineral contents in *M. vulgare*.

Harvest Site	Minerals (ppm) *									
	Ca	Mg	Fe	Zn	Cu	Mn	K	Pb	Cd	Ni
Boussalem	38,720 ^a^ ± 4	9648 ^a^ ± 2	208 ^ab^ ± 1	620 ^a^ ± 0	55 ^b^ ± 0	7 ^ab^ ± 0	20,800 ^b^ ± 0	0	11 ^a^ ± 0	34 ^ab^ ± 0
Bizerte	52,352 ^a^ ± 4	128,40 ^a^ ± 2	2848 ^ab^ ± 1	1124 ^a^ ± 0	26 ^a^ ± 0	5 ^a^ ± 0	112,00 ^a^ ± 0	0	13 ^a^ ± 0	28 ^ab^ ± 0
Tunis	46,160 ^a^ ± 1	12,448 ^a^ ± 5	2664 ^ab^ ± 1	869 ^a^ ± 0	28 ^a^ ±0	33 ^b^ ± 0	15,600 ^ab^ ± 0	0	12 ^a^ ± 0	122 ^b^ ± 0
Zaghouan	55,760 ^a^ ± 31	8176 ^a^ ± 0	1313 ^a^ ± 0	531 ^a^ ± 0	18 ^a^ ±0	7 ^ab^ ± 0	8400 ^a^ ± 0	0	12 ^a^ ± 0	28 ^ab^ ± 0
Kasserine	54,312 ^a^ ± 21	7672 ^a^ ± 0	9104 ^b^ ± 6	1148 ^a^ ± 0	24 ^a^ ±0	28 ^b^ ± 0	14,400 ^ab^ ± 0	0	8 ^a^ ± 0	22 ^a^ ± 0

* Each value represents the mean ± SD (*n* = 3). Means followed by the same letter are not different according to ANOVA (analysis of variance) (* *p* < 0.05), a > b.

**Table 6:** Composition of *M. vulgare* essential oil collected at different locations in Tunisia.

Compound	Rt (min)	Leaves				Stems			
		Bizerte	Boussalem	Zaghouan	Tunis	Bizerte	Boussalem	Zaghouan	Tunis
Linalool	4.13	-	-	1.05	0.49	-	-	0.36	0.13
Phenylethyl alcohol	4.30	-	-	-	0.16	-	-	-	0.10
1-terpinol	4.52	0.12	-	-	-	-	-	-	-
Ketoisophorone	4.54	-	-	-	0.21	-	-	-	-
Indole	5.84	-	1.17	0.25	2.11	0.20	0.27	-	0.24
2-methoxy-4-vinylphenol	5.94	-	1.72	1.51	-	0.31	0.42	0.49	-
3-allyl-6-methoxyphenol	6.26	-	-	-	-	0.69	-	-	-
Eugenol	6.28	-	15.29	0.98	8.06	-	2.18	0.27	1.31
Trans-caryophyllene	6.83	-	-	-	0.61	-	-	-	-
Geranylacetone	6.90	-	0.75	-	-	-	-	-	-
β-ionone	7.22	-	4.14	-	-	-	-	-	-
Germacrene D	7.25	-	-	1.34	-	-	-	0.60	-
β-bisabolene	7.35	-	2.37	-	1.60	0.42	0.4	-	3.14
δ-cadinene	7.49	-	-	1.71	1.07	-	-	-	-
Dihydroactinidiolide	7.64	-	-	-	1.41	-	0.70	-	-
Nerolidol	7.67	-	1.02	-	-	-	0.5	-	-
Hexadecame	7.85	-	-	-	-	27.29	-	-	-
Megastigmatrienone 2	7.86	-	2.45	-	1.95	-	0.49	-	-
Spathylenol	7.94	-	-	1.48	-	-	-	-	-
Caryophyllene oxide	7.99	-	0.70	0.65	0.56	-	-	-	-
Megastigmatrienone isomer	8.19	-	1.68	-	-	-	0.27	-	-
τ muurolol	8.31	-	-	-	-	-	-	1.24	-
τ cadinol	8.32	-	-	4.40	-	-	-	-	-
α-cadinol	8.40	-	-	-	-	-	-	1.01	-
Miristic acid	8.88	-	-	-	0.84	0.42	-	-	-
Hexahydrofarnesyl acetone	9.36	-	1.40	0.78	1.06	-	0.28	-	-
Methyl palmitate	9.80	1.23	-	-	-	-	-	-	-
Palmitic acid	10.04	-	-	-	-	9.99	2.03	0.57	2.00
Methyl linoleate	10.75	-	-	-	-	-	1.35	-	-
Phytol	10.81	-	1.04	-	-	-	-	-	-
Linoleic acid	11.01	-	-	-	-	3.27	-	-	-
Butyl palmitate	11.14	10.95	1.09	-	0.74	-	-	-	-
Tricosane	11.68	-	-	-	-	-	1.35	-	-
Tetracosane	12.14	-	-	-	-	-	1.95	-	-
Pentacosane	12.61	-	-	-	-	-	4.05	-	0.99
Hexacosane	13.01	-	-	-	-	-	4.77	-	0.45
Heptacosane	13.63	-	-	-	-	-	5.49	-	2.04
Eicosane	13.63	2.63	-	-	-	-	-	-	-
Octacosane	14.24	-	-	-	-	-	5.45	-	2.21
Squalene	14.54	-	-	-	-	-	-	1.85	-
Marrubin	14.64	-	4.09	-	-	-	-	6.67	-
Nonacosane	14.95	-	-	-	-	-	4.67	-	2.48
Triacontane	15.82	-	-	-	-	-	4.27	-	2.76
Terpenoids		0.12	18.6	11.41	8.96	0.42	2.64	11.73	3.27
Other compounds		14.81	20.31	2.74	11.07	41.75	38.25	1.33	15.37

**Table 7:** Toxicity (% of mortality) of essential oils from *M. vulgare* against Artemia salina *.

Harvest Site	Essential Oil (2% *v*/*v*)	
	Leaves	Stems
Bizerte	7.14 ^a^ ± 1.00	9.52 ^a^ ±1.52
Boussalem	16.66 ^c^ ± 0.58	14.28 ^b^ ± 2.00
Zaghouan	ND	19.04 ^c^ ± 1.00
Tunis	11.90 ^b^ ± 0.57	14.28 ^b^ ± 0.00

* Each value represents the mean ± SD (*n* = 3); ND: Not determined; means followed by the same letter are not different according to ANOVA (analysis of variance) (* *p* < 0.05), a > b > c.
